# The Effect of Fatigue on Kicking Velocity in Soccer Players

**DOI:** 10.2478/v10078-012-0083-8

**Published:** 2012-12-30

**Authors:** Ricardo Ferraz, Roland van den Tillaar, Mário C. Marques

**Affiliations:** 1 Department of Sports Sciences, University of Beira interior, Covilhã, Portugal.; 2 Research Centre for Sport, Health and Human Development, Portugal.; 3 Department of Teacher Training of Nord Trøndelag University College, Norway.

**Keywords:** kicking velocity, soccer, fatigue, general governor model

## Abstract

Soccer is a game in which fatigue can negatively influence players’ performance. Few studies have examined the practical effects of fatigue on soccer performance skills. Thus, the aim of the present study was to evaluate the effect of fatigue, acutely induced by means of a soccer specific circuit on ball velocity. Ten amateur soccer players (age 27.3 ± 5.25 yr; experience 16,8 ± 6.05 yr; level secondary division; body height 1,80 m ± 0,06; body mass 75,7 kg ± 5,78), participated in this study and performed maximal instep kicks before and after the implementation of an intensive, intermittent and repeated exercise protocol. Analysis of variance with repeated measures indicated a significant decrease (p<0.05) in ball velocity after just one round of the fatigue circuit. However, after the third circuit ball velocity increased and after the fifth circuit maximal ball velocity increased yet again (compared to the second circuit) and was not significantly different from before commencement of the fatigue protocol. The results partly confirmed the hypothesis of the negative influence of fatigue upon ball velocity in soccer kicking, demonstrating also some variability in the presented values of ball velocity perhaps theoretically accounted for by the general governor model.

## Introduction

The game of soccer requires intermittent efforts with activity changes every 3–5 seconds, characterised by alternating moments of high intensity and of almost complete rest (Bangsbo, 1994). High intensity moments are those which normally correspond to decisive actions, i.e., scoring a goal. Kicking performance is perhaps the most important action in soccer since it supports a key objective of the game: scoring goals by kicking the ball.

Due to this inherent importance, the study of kicking in soccer has raised scientific interest, which has resulted mainly in studies from the point of view of biomechanics, technical analysis, muscular involvement in the kicking action and even studies in footwear (Forestier and Nougier, 1998; Madigan and Pidcoe, 2003; Sterzing, 2010). These studies show that there are many factors that can determine the action of soccer kicking, such as body posture, technical approach, footwear, muscle strength and power output and fatigue. However, the precise influence of fatigue on soccer kicking has not yet been clarified.

Fatigue is manifested by a reduction of maximal force or power that is associated with sustained exercise and is reflected in a decline of performance (Mohr et al., 2002). It has been noted that players experience fatigue both towards the end of a game and temporarily over its duration (Kellis et al., 2006). Fatigue experienced during a soccer game is manifested by a reduced capacity to perform the critical actions of high intensity mentioned above, along with a progressive reduction of muscle strength. Thus, the ability to resist the negative effects of fatigue is a key factor for a soccer player. Fatigue can be considered as a performance constraint that affects the motor processing and perceptual processing that is linked to the performing skills required in game situations (McMorris and Graydon, 1997).

The negative effect of fatigue may be due to a neuromuscular decrease in performance induced by acute and immediate effort. This is probably caused by changes in muscle strength and coordination due to physiological causes and inherent metabolic changes (Kellis et al., 2006; Mohr et al., 2005).

Some studies have found significant effects of local muscle fatigue protocols on the performance of complex discrete movements as in handball throwing (Forestier and Nougier, 1998) and vertical jumping (Rodacki et al., 2001). Others, using fatigue protocols related to long-distance running, have reported a significant decline in leg power, maximum isometric force and activity of the quadriceps (Nicol et al., 1991) as well as alterations in circuit reaction force (GRF) and joint kinematics of running (Madigan and Pidcoe, 2003; Mizrahi et al., 2000).

Although fatigue is considered an important factor in soccer as well as in soccer skills proficiency (Mohr et al., 2005; Stone and Oliver, 2009) and despite the fact that player kicking ability is seen as one of the most important determinants of soccer performance (Rampinini et al., 2009; Russell et al., 2011), few studies have examined the effect of fatigue on ball velocity in the soccer kicking performance. Some studies have tried to explain the effect of fatigue on physical conditioning variables related to biomechanical and muscular analysis in the lower limbs based on the effects of prolonged intermittent specific soccer exercises. It was found that after fatigue an increased electro-mechanical delay and knee joint laxity occurs together with a significant decline of maximum isokinetic moment of force of both knee extensors and flexors (Drust et al., 2000; Rahnama et al., 2003). It was also found that fatigue developed during a match after relatively intense intermittent activities negatively affected the short-passing performance ability as shown by the increased number of errors made during the test and the time required to perform the test (Rampinini et al., 2008).

With respect to the studies considering the soccer kicking performance most of them occur in conditions with no fatigue as a variable (Lees and Davies, 1988). Only three studies have investigated the effect of fatigue on kicking following intermittent exercise protocols. Kellis et al. (2006) found a decline in kicking ball velocity following a fatigue protocol. However, Currell et al. (2009) reported that ball velocity performance in soccer kicking did not change during simulated match play and Russell et al. (2011) also found no evidence that fatigue affects average ball velocity in soccer kicking. However, Russell et al. (2011) did conclude that peak kicking velocity tends to reduce in the second half of a soccer match simulation protocol (including passing, dribbling and shooting skills). For all three studies, the results are variable and inconclusive. There is evidence which shows the effect of fatigue on biomechanical and muscular performance but the practical effects on soccer skills performance and soccer kicking are still unclear. In our view, the protocols used may limit the validity of the results as they were based on few measurements of ball velocity. Kellis et al. (2006) took measurements at three intervals (before induction of fatigue; through the protocol; and at the end of the induced fatigue protocol) and Russell et al. (2011) took measurements before and after the first and second part. For a proper understanding of the temporal changes, a longer test period seems essential to understand what really happens to kicking ball velocity under the influence of fatigue.

The aim of this study was therefore to investigate the influence of fatigue upon maximal ball velocity in soccer kicking. It was hypothesized that acutely induced fatigue has a negative influence on ball velocity i.e. that peak ball velocity decreases with increasing levels of induced fatigue. It is also important to understand how this influence is expressed over the period of an intermittent and specific exercise protocol application and whether or not the effect is progressive.

## Material and Methods

### Experimental Approach to the Problem

A repeated-measures design with one group of amateur soccer players was used to determine the influence of acutely induced fatigue on ball velocity in kicking. Fatigue was induced by a soccer specific circuit performed for 90 s five times.

### Subjects

Ten amateur soccer players (age 27.3 ± 5.25 yr; experience 16,8 ± 6.05 yr; level secondary division; body height 1,80 m ± 0,06; body mass 75,7 kg ± 5,78), participated in this study. Participants were fully informed about the protocol before the experiment. Informed consent was obtained prior to testing from all subjects, according to the approval of the local ethics committee and current ethical standards governing sports and exercise research.

### Procedure

**Figure 1:**
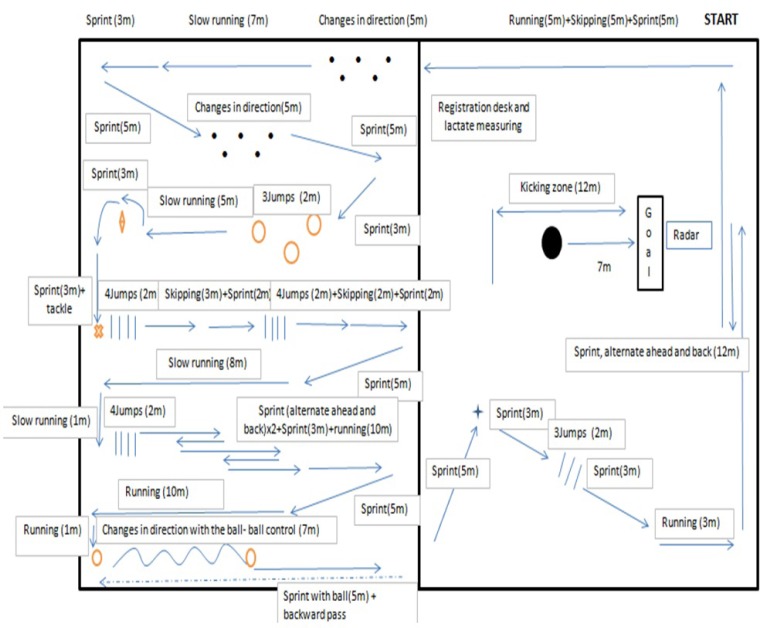
Circuit Design

After a general warm-up of 15 minutes which included jogging and kicking drills, ball velocity was tested from 7 m (“penalty kick”). A standard soccer ball (mass approximately 430 g, circumference 70 cm) was used. The instruction was to kick a regular ball with maximum force and attempt to hit a target from seven meters distance, aiming at a 1 by 1 m circled target at 2 m height located in the middle of a goal (3 × 2 meters). Three attempts in each case were made. Immediately afterwards, the subjects performed the soccer specific circuit (Figure 1) involving high intensity activities. The circuit consisted of a set of specific and explosive exercises including jumps, skipping, multiple changes of direction, dribbling the ball, passing, bursts of sprinting and jogging (Figure 1). After following the circuit for 90 seconds, the participants had to kick the ball a further three times, followed by 90 seconds of rest before the start of the next 90 s circuit. Subjects repeated the circuit 5 times. If a given participant completed the circuit in under 90 seconds, he continued to follow it until the set time was reached.

### Measurements

Maximal ball velocity was determined using a Doppler radar gun (Sports Radar 3300, Sports Electronics Inc.), with ± 0.028 m·s^−1^ accuracy within a field of 10 degrees from the gun. The radar gun was located 1 m behind the goal at ball height.

The highest ball velocity of all three attempts after each 90 seconds circuit was used for further analysis together with average ball velocity and standard deviation to discover whether variability in ball velocity increased. The total distance covered during the 90 seconds of the circuit was also measured. This measurement represented the sum of the meters previously marked along the circuit rounded up to the nearest meter. Participants wore a heart rate monitor (Polar, RS300x) for the duration of the experiment. Heart rate was measured immediately following the completion of each circuit and just before the start of the next, together with the rating of perceived exertion (RPE) on a 20 points Borg scale (Borg, 1973). Lactate was measured after the warm up and directly after the three kicks following each circuit. Blood was taken from the fingertip and lactate measurement was performed using a portable apparatus (Roche Accutrend Lactate Test Strips, Basel, Swiss).

### Statistical analyses

To assess differences in maximal ball velocity, heart rate, lactate, RPE and total meters covered after completion of the circuits, a repeated analysis of variance (ANOVA) design was used. Least significant differences (LSD) analyses were conducted to locate differences. All results are presented as mean ± SD. Where sphericity assumption was violated, the Greenhouse-Geisser adjustments of the p-values were reported. The criterion level for significance was set at p<0.05. Effect size was evaluated with η^2^_p_ (Eta partial squared) where 0.01< η^2^<0.06 constitutes as a small effect, a medium effect and when 0.06< η^2^<0.14 and a large effect when η^2^>0.14. Statistical analysis was performed in SPSS version 18.0 (SPSS, Inc., Chicago, IL).

## Results

**Figure 2:**
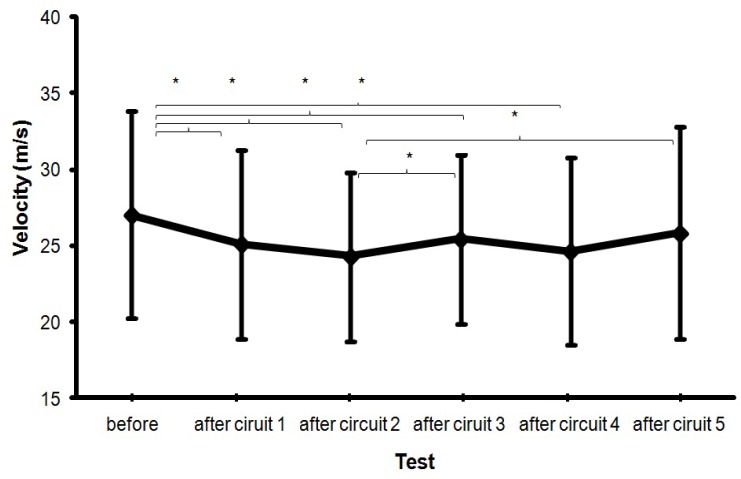
Maximal ball velocity (±SD) before and after conducting each circuit (m/s) * indicates a significant difference between these two ball velocities on a .05 level.

**Figure 3:**
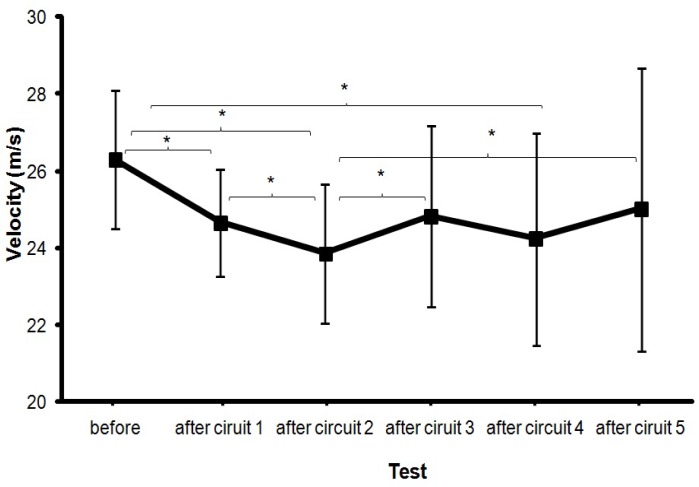
Average ball velocity (±SD over the three attempts averaged per subject) before and after conducting each circuit (m/s) * indicates a significant difference between these two ball velocities on a .05 level.

The Oneway ANOVA showed that the maximal ball velocity was affected significantly after completion of the circuit (*F* = 7.6, p<.001, η^2^ = 46). Post hoc comparisons showed that the ball velocity decreased significantly after just one circuit of the fatigue protocol compared with the ball velocity before the start of the circuit. However, after circuit 3, ball velocity increased (compared to circuit 2) and after circuit 5, maximal ball velocity increased yet again (compared to circuit 2) and was not significantly different from before the start protocol (Figure 2). When average ball velocity was measured a significant decrease was found after circuit 1 and after circuit 2. From circuits 2 to 3 an increase was found, and again after circuit 5 (*F* = 4.3, p=.003, η^2^ = .32; Figure 3).

**Figure 4:**
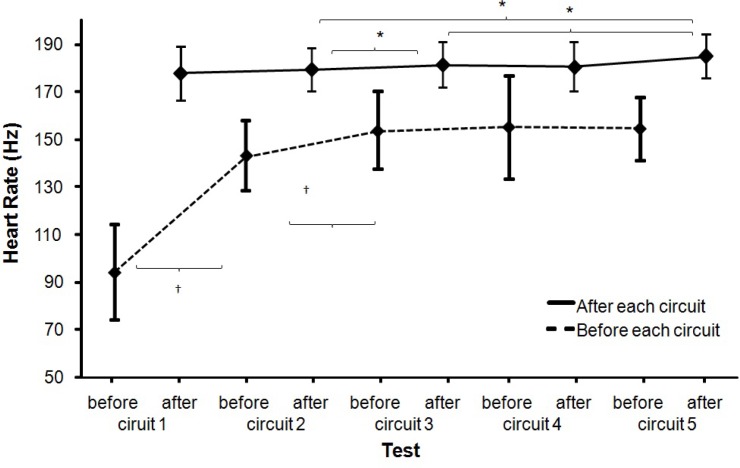
Mean (±SD) heart rates before the start of each circuit and after each circuit. * indicates a significant difference on a .05 level between these two conditions. ^†^ indicates a significant difference on a .05 level between these two conditions and all right for this.

**Figure 5:**
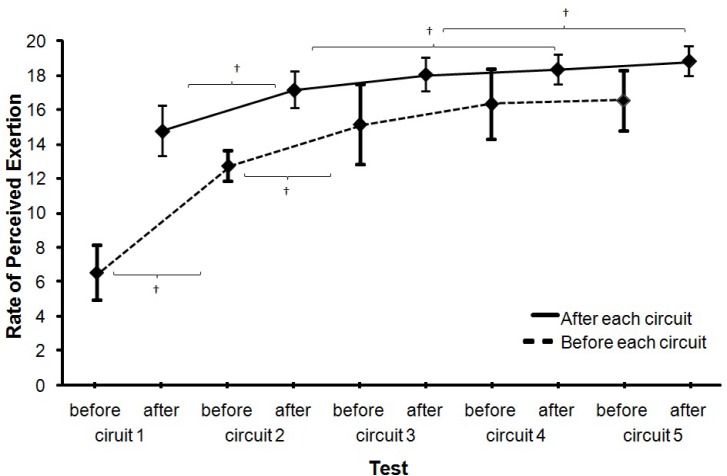
Mean (±SD) rate of perceived exhaustion before and after each circuit. * indicates a significant difference on a .05 level between these two conditions. ^†^ indicates a significant difference between these two conditions and all right for that.

Heart rate as measured before the start of each fatigue circuit (*F* = 57.5, p<.001, η^2^ = .87) increased significantly over the exercise period, but not significantly after each circuit (*F* = 2.0, p=.120, η^2^ = .18). This was accompanied by an increase in the rate of perceived exertion (*F* ≥ 45.8, p<.001, η^2^ ≥ .73; Figure 4 and 5). Post hoc comparison revealed that the heart rate before the start of each fatigue circuit significantly increased only until the start of circuit 3, while heart rate after each circuit significantly increased from circuits 2 to 3 and after the last circuit significantly (Figure 4). The rate of perceived exertion (RPE) before the start of each circuit also increased significantly only up to the start of circuit 4, while the RPE after each circuit significantly increased from 1 to 2, 2 to 4 and from 3 to 5 (Figure 5).

**Figure 6:**
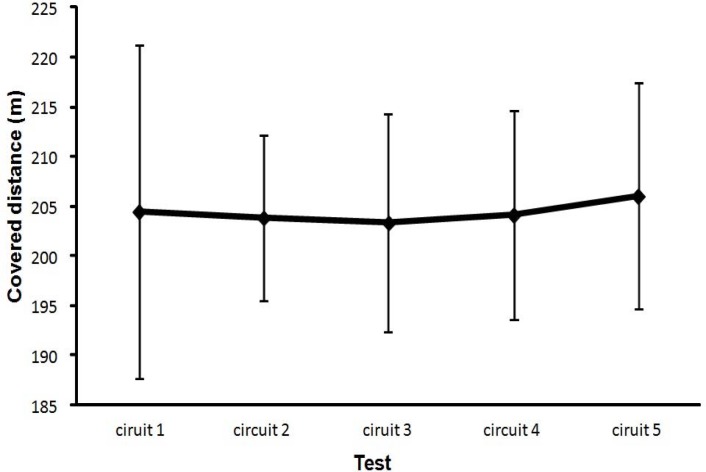
Mean (±SD) distance covered after ending each circuit.

However, the fatigue associated with the completion of the circuit was not shown in measures of meters covered during the ninety-seconds of the circuit. Distance covered was almost the same after each circuit (*F* = 0.1, p = .83, η^2^ = .006, Figure 6).

**Figure 7:**
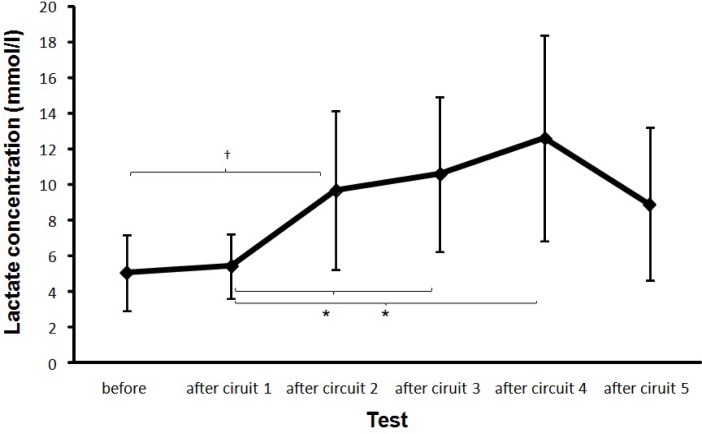
Mean (±SD) initial lactate concentration and measured after each circuit. * indicates a significant difference on a .05 level between these two conditions. ^†^ indicates a significant difference between these two conditions and all right for these conditions

Lactate concentration changed significantly during the protocol (*F* = 4.9, p = .002, η^2^ = .41). Post hoc comparison showed that lactate concentration only increased significantly after completion of the second 90 s fatigue circuit. Also a significant increase in lactate concentration after the first circuit compared to the third and fourth circuit was found (Figure 7).

## Discussion

The main finding was that the maximal and average ball velocity decreased after completion of a fatigue protocol. However, after just two circuits of this protocol ball velocity showed no further decline (Figure 1 and 2), while heart rate, RPE and lactate concentration as measures of fatigue continued to rise after the second circuit.

The results confirm, in part, the hypothesis of the negative influence of fatigue upon ball velocity in soccer kicking, demonstrating also some variability in the presented values of ball velocity. The decrease in ball velocity at the termination of all repeated circuits with the one before the start of the first circuit (with exception of the last one) is consistent with results found in other studies (Kellis et al., 2006; Russell et al., 2011).

In these studies it was confirmed that fatigue had a negative influence on ball velocity in soccer kicking (Kellis et al., 2006).

Possible explanations can be used from a broad range of biomechanical and physiological analyses such as: decreased neuromuscular performance; changes in the pattern of muscle strength; changes in coordination due to inherent physiological causes (Ismail et al., 2010; Seyed et al., 2010) changes in the moment of force (as shown in velocity) of the leg before ball contact; and consequent decrease in strength of the muscles involved in kicking.

Other explanations such as approach speed and a skill level (Lees and Nolan, 1998) have been suggested in previous studies as responsible causes for the lower velocity as transferred to the ball (Kellis et al., 2006). Along with those already cited, Ekblom (1986) showed that the blood lactate level and decreased muscle glycogen are connected to impaired neuromuscular performance affecting coordination and consequently soccer performance skills. However, the results of the present study following completion of the third circuit show that these explanations are inadequate.

These results need to be treated with some caution. Taking into account the related literature, it was expected that ball velocity would reduce progressively down to a given limit. However, the results show that after three circuits, ball velocity started to increase again (Figure 2 and 3), even though heart rate, RPE and lactate concentration continued to increase. After completion of the last repetition of the circuit (circuit 5) there was, in fact, no significant difference in maximal ball velocity as compared to the initial ball velocity measured before the start of the first circuit (Figure 2). Even when the player started and finished each circuit considerably more fatigued, as shown by the increased RPE and heart rate (Figure 4 and 5), he was able to attain a similar distance in meters covered (Figure 6) and after two circuits the ball velocity in soccer kicking started to increase again (Figure 2). These findings seem counterintuitive and no real explanation for them exists in any related study. However, we consider that the phenomenon may best be explained by the central governor model and the concept of “pacing” (Noakes et al., 2004; St Clair Gibson and Noakes, 2004; Lambert et al., 2005; Noakes et al., 2005).

The central governor model is a theory developed by Noakes (Noakes et al., 2004; St Clair Gibson and Noakes, 2004; Lambert et al., 2005; Noakes et al., 2005) which explains the phenomenon of fatigue. It proposes that physical activity is controlled by a central governor in the brain and that the human body functions as a complex system during exercise. The subconscious brain regulates power output - pacing strategy - by modulating motor unit recruitment to preserve whole body homoeostasis and prevent catastrophic physiological failure (Noakes et al., 2004; St Clair Gibson and Noakes, 2004; Lambert et al., 2005; Noakes et al., 2005).

According to the theory, exercise intensity and the activity of different metabolic systems oscillate continuously as a result of multiple interactions between all the organs that contribute to this complex system.

In our study an increased index of fatigue (Figure 5) was recorded (increase of RPE over the 5 circuits). However, fatigue negatively influenced ball velocity only in the first part of the fatigue protocol and not in the same way in the second part.

Millet’s flush model (Millet, 2011), based on the principles of the governor model, explains the regulation of fatigue in endurance activities specifically adapted to ultra endurance running by citing the role of motivation and what he calls a security reserve. According to the flush model, there is always a reserve for muscle recruitment (the security reserve) that can be used for the so-called “end spurt” at the highest level of peripheral fatigue. A capacity to increase acceleration close to the finish of ultra marathon running in face of decreasing energy was found to be an effect of mental motivation. Thus, although the RPE increases over longer distance and running velocity at first decreases, velocity is recuperated at the end due to increased motivation (as the runner approaches the finishing line) and/or a recruitment from the security reserve.

We observed the same principles in our study. A decline in ball velocity was followed by an increase in ball kicking velocity after the third circuit. We assume that this was possible due to muscle recruitment from the security reserve and/or an increase in motivation arising from the subjects’ awareness that they were nearing the end. In the final circuit, ball speed reached similar values to those measured at the start, perhaps because subjects knew that this was really the last time and that they could therefore use (consciously or not) the security reserve. This was also shown by a slight increase in distance covered (Figure 6), heart rate (Figure 4) and a slight fall in lactate concentration (Figure 7). After the final circuit, the higher heart rate and the increased distance covered indicated higher energy use by subjects, reinforced by the fact that lactate levels also fell slightly. This fall in lactate concentrate was probably due to its use as a fuel; since it has been found that, during high intensity exercise, substrates other than glucose (such as lactate) may contribute significantly to cardiac energy production (Chatham, 2002).

The concepts as “security reserve” and “pacing strategy” seem to have relevance in our study and we can also find reasons in literature specifically related with soccer players. According to Edwards and Noakes (2009) soccer players are well known to self-regulate match-play efforts following numerous intrinsic and extrinsic factors. They suggested that players modulate effort according to a subconscious strategy. As such, subconscious physiological factors influence conscious behavioral decisions to regulate effort. Also, fatigue seems to affect pacing strategies of the players (Orendurff et al., 2010). Consequently as shown in our study we suggest that players may have self regulated their effort (pacing strategy) throughout the protocol. Since they knew the number of repetitions of the circuit the players could pace themselves and perform better in the last repetition due to the fact that they knew that it was their last time. Future studies involving this knowledge about length of a fatigue protocol should be conducted to investigate this effect of knowledge upon a possible pacing strategy in soccer kicking.

## Practical Applications and Conclusions

Our study demonstrated and strengthened the hypothesis concerning the potential negative effects of fatigue on kicking in soccer and showed in addition that the effect of fatigue can be variable, i.e. neither linear nor progressive. The reported findings can be related to the effect of fatigue on kicking soccer theorised according to the central governor model as a holistic and complex approach. The “security reserve” and the associated mental/motivational concept as “pacing” may have significant explanatory potential. Therefore, practitioners should be aware that fatigue influences ball velocity in kicking as during soccer games. However, this performance can be variable and maybe dependent on self-regulation of the effort of the players according to some conscious and subconscious factors. However, future studies need to be conducted to confirm and understand the influence of fatigue from different “pacing strategies” in close actions as a kicking but also in broader contexts such as training and during competition.
